# Supplementary material for: Speech-induced suppression during natural dialogues
Source: Commun Biol. 2024 Mar 8;7:291. doi: 10.1038/s42003-024-05945-9 (PMC10923813; doi:10.1038/s42003-024-05945-9)
Supplement: Supplementary file 2 — Suplementary Information [file 42003_2024_5945_MOESM2_ESM.pdf]

Supplementary Information of Speech-induced suppression during natural dialogues

Joaquin E. Gonzalez[1,*], Nicolás Nieto[2,3], Pablo Brusco[4], Agustin Gravano[5,6,7], and Juan E. Kamienkowski[1,4,8]

[1]Laboratorio de Inteligencia Artificial Aplicada, Instituto de Ciencias de la Computación (Universidad de Buenos Aires - Consejo Nacional de Investigaciones Cientificas y Tecnicas), Buenos Aires, Argentina

[2]Instituto de Investigación en Señales, Sistemas e Inteligencia Computacional, sinc(i) (Universidad Nacional del Litoral - Consejo Nacional de Investigaciones Cientificas y Tecnicas), Santa Fe, Argentina

[3]Instituto de Matemática Aplicada del Litoral, IMAL-UNL/CONICET, Santa Fe, Argentina

[4]Departamento de Computación, Facultad de Ciencias Exactas y Naturales, Universidad de Buenos Aires, Buenos Aires, Argentina

[5]Laboratorio de Inteligencia Artificial, Universidad Torcuato Di Tella, Buenos Aires, Argentina

[6]Escuela de Negocios, Universidad Torcuato Di Tella, Buenos Aires, Buenos Aires, Argentina

[7]Consejo Nacional de Investigaciones Científicas y Técnicas, Buenos Aires, Argentina

[8]Maestria de Explotación de Datos y Descubrimiento del Conocimiento, Facultad de Ciencias Exactas y Naturales - Facultad de Ingenieria, Universidad de Buenos Aires, Buenos Aires, Argentina

[*]joaquin.gonzalez6693@gmail.com

Supplementary Note 1: Data synchronization

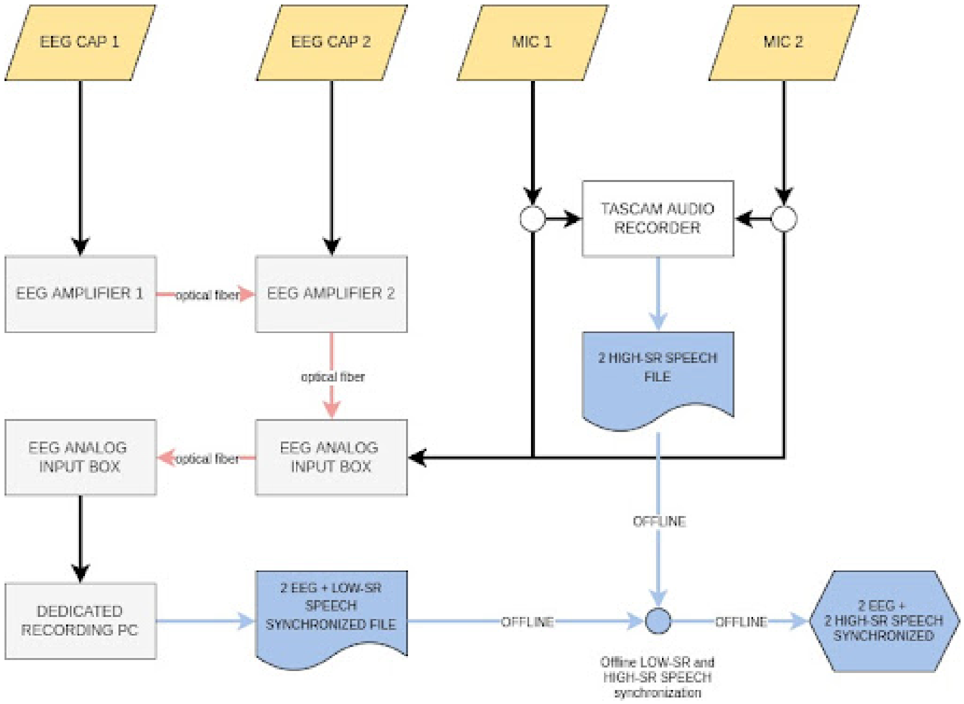

Supplementary Figure 1: Recording and synchronization of EEG and audio channels.

Supplementary Note 2: Lateralization

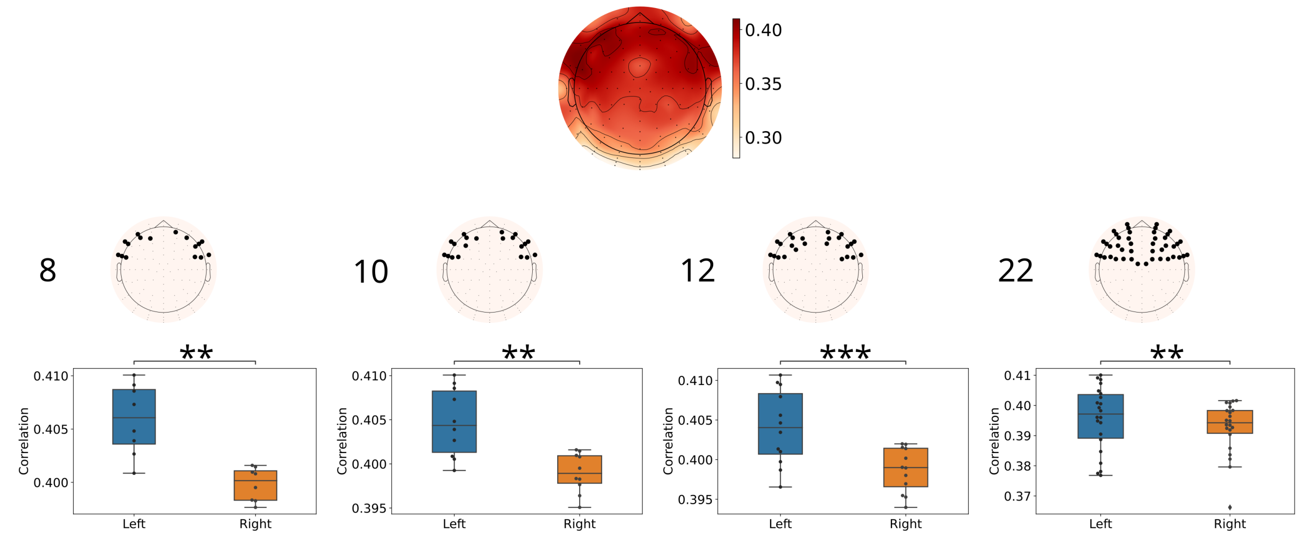

Supplementary Figure 2: Correlation distribution for left and right electrodes indicated in the topographic figure, for the spectrogram model for 8, 10, 12, and 22 selected electrodes. The electrodes were chosen in each case, as the ones presenting higher correlation values in the frontal region for each hemisphere. A signed-rank Wilcoxon test was performed to compare the values obtained in each hemisphere. The correlation values for the spectrogram show a significant lateralization effect towards the left hemisphere in all cases. Significance: ** p-value $<$ 0.01, *** p-value $<$ 0.001..

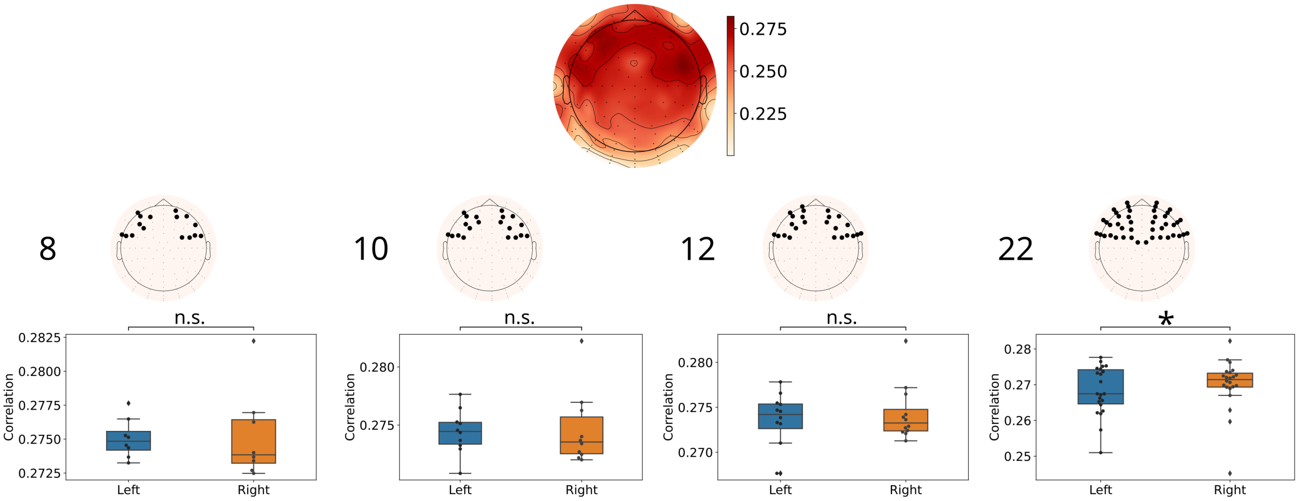

Supplementary Figure 3: Correlation distribution for left and right electrodes indicated in the topographic figure, for the envelope model for 8, 10, 12, and 22 selected electrodes. The electrodes were chosen in each case, as the ones presenting higher correlation values in the frontal region for each hemisphere. A signed-rank Wilcoxon test was performed to compare the values obtained in each hemisphere. The correlation values for the envelope show no significant lateralization effect when considering the higher correlation values, but it does when considering all electrodes in the frontal lateral region. Significance: n.s. p-value $>$ 0.05, * p-value $<$ 0.05.

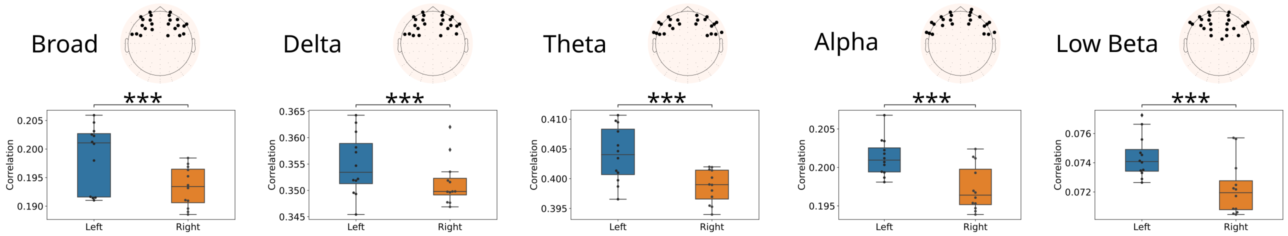

Supplementary Figure 4: Correlation distribution for the left and right electrodes indicated in the topographic figure, using the spectrogram as input for the model, repeated for all frequency bands. The electrodes were chosen in each case as the 12 presenting higher correlation values in the frontal region for each hemisphere. A signed-rank Wilcoxon test was performed to compare the values obtained in each hemisphere. The correlation values for the spectrogram show a significant lateralization effect towards the left hemisphere in all cases. Significance: *** p-value < 0.001.

Supplementary Note 3: Other features

The voice Pitch was calculated using Praat software,[1] through a Python library (Praatio). Limit values for pitch computation were set for men between 50 Hz and 300 Hz and for women between 75 Hz - 500 Hz, and the silence threshold value for pitch calculation was set to 0.03 of the maximum amplitude of the audio signal. The resulting pitch for each participant was then downsampled to 128 Hz similarly to the other variables. It should be noted that in the speech samples, there are moments of silence, either between or within words, and in those moments pitch is not defined. For this reason, and in order to perform an analysis like the one implemented with the audio envelope (which is always defined), the missing values were completed with 0. In contrast with our results, previous studies that explore the representation of speech acoustic features in the brain found a relatively higher importance of the pitch. But, those studies were performed in English where there is a stronger connection between pitch-accent and discourse meaning than in Spanish. Instead, the latter uses word order to express information structure and to convey discourse prominence.[2]

The shimmer represents the amplitude variation of the audio signal over time. It was computed using Praat software, through Parsemouth Python library.[3]

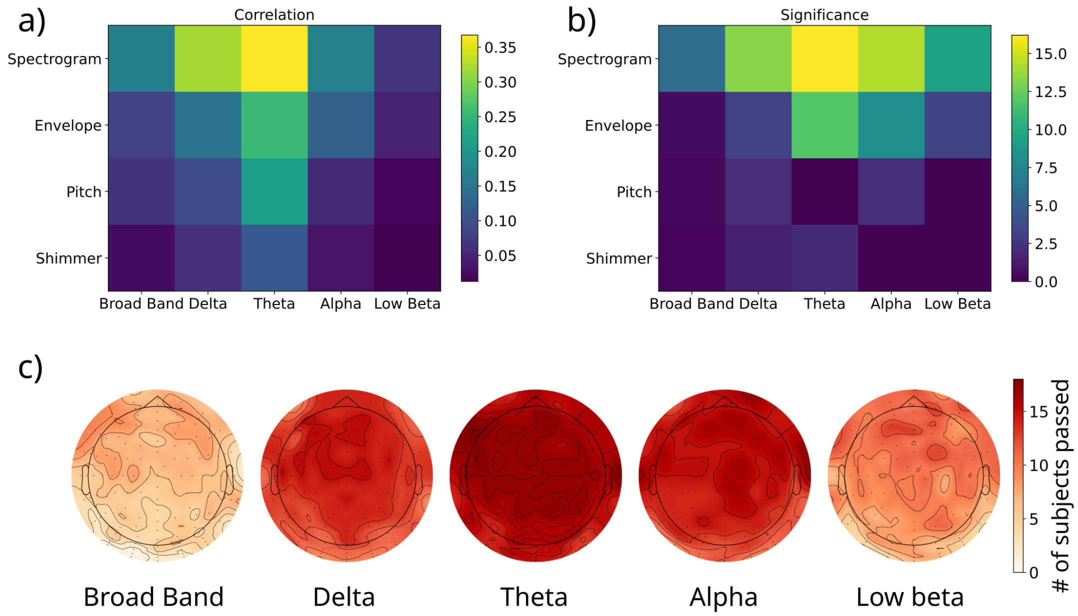

Supplementary Figure 5: Summary heatmaps for the correlation values (A) and the statistical significance (B) averaged across channels, for each frequency band and feature. (C) Spatial distribution of model significance across participants for every frequency band of the spectrogram feature, corresponding to the first row of panel B. A permutation test was applied to each electrode, fold, and participant (see Section 4.7.1). An electrode was considered to have a significant effect if its correlations passed the test in all the folds. The scalp distributions show the number of participants with significant results for each electrode. The maximum possible value was 18, as the number of participants.

Supplementary Note 4: Pre-stimulus onset

Figure 6 shows the mTRFs as a traditional ERP response. For this analysis, we used the same 0.6 s for the main analysis, but predicted the EEG sample corresponding to 0.4 s instead. In that way, the audio feature samples of the last 0.2 s were pronounced after the time point of the EEG signal that we're aiming to predict, and thereby could have no causal connection. As the time axis represents the time elapsed between the pronunciation of the audio features and the time point from the EEG signal being predicted, such timelags correspond to the negative values in the axis. As expected, we can see that those time lags show no response in the EEG to the input feature, except for around 20 ms before the time 0. This can be explained by the fact that the contiguous samples of the audio envelope, and hence the audio features, have a temporal correlation to one another, making the model capture a response to a temporal time lag because of the similarity to the samples of that feature after the onset time.

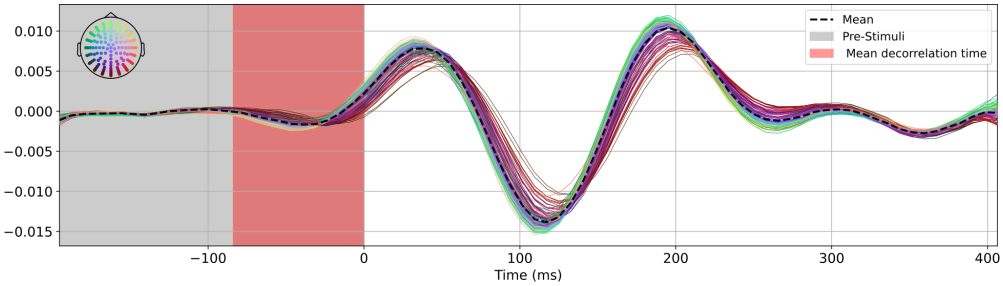

Supplementary Figure 6: mTRFs as ERP with negative timelags. The shaded grey times correspond to time lags with no causal connection to the EEG signal. In red, is the mean decorrelation time of the Envelope of the listened audio signal.

Supplementary Note 5: Signal filters

As explained in the Methods section, the filters applied to band-pass filter the EEG signals were causal FIR filters using the window method.[4] The parameters were set to use a 'hamming' window, to pad the edges with the signal edge values, and to introduce a 'minimum' phase lag in the causal filter. The Hamming window width was 0.0194s pass-band ripple and 53 dB stop-band attenuation.

The reason why minimum phase filters were used is that non-causal zero-phase filters would modify the temporal causality in the EEG signal, which would have considerable and undesirable implications in the mTRF fitting and timing. According to,[5] the mTRF results from causal and non-causal filters only slightly differed by delaying the response around 50 ms for causal filters. In our case, we had approximately a 100 ms difference between the two filters (see below). Moreover, the linear phase filter and the causal filter presented opposed polarisations in the mTRFs, where the causal filter showed results agreeing with the previous literature.[6–8]

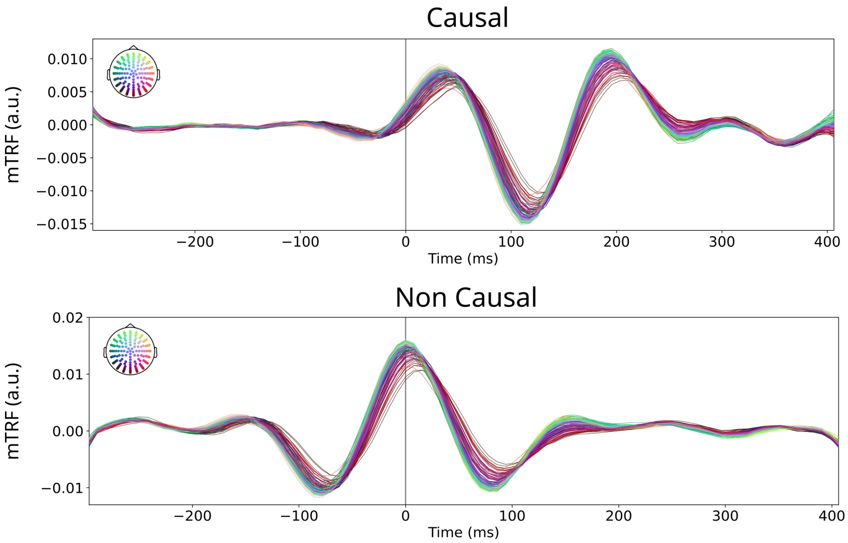

Supplementary Figure 7: Average mTRF of all participants fitted using spectrogram features as input in the Theta band from -300 ms to 400 ms as target. The top panel shows the mTRF when the EEG signal was filtered using a causal filter. The lower panels show the same procedure when using non-causal zero-phase filters.

	Delta	Theta	Alpha	Low Beta	Broad
Lower transition (Hz)	1	2	2	3.25	-
Upper transition (Hz)	2	2	3.25	3.4.75	10
Filter length (samples)	1691	845	845	521	169

Supplementary Table 1: Filter parameters.

Effect on results

To assess the robustness of the results and to compare them with previous work, we repeated the analysis using combinations of dialogue conditions instead of each condition separately. This way, the "Silence" intervals would also be considered in the encoding model, as is the case in most of the previous work.

Filter	Frequency band	Condition	Correlation
FIR	Theta	Listening (E)	0.367 ± 0.032
FIR	Theta	Speaking (S)	0.020 ± 0.005
Butterworth	Theta	All listening: (E) + (E|B) + Silence	0.333 ± 0.029
Butterworth	Theta	All speaking: (S) + (S|B) + Silence	0.014 ± 0.005
FIR	Theta	All listening: (E) + (E|B) + Silence	0.308 ± 0.029
FIR	Theta	All speaking: (S) + (S|B) + Silence	0.013 ± 0.005
Butterworth	1 - 8 Hz	All listening: (E) + (E|B) + Silence	0.301 ± 0.027
Butterworth	1 - 8 Hz	All speaking: (S) + (S|B) + Silence	0.022 ± 0.005
FIR	1 - 8 Hz	All listening: (E) + (E|B) + Silence	0.276 ± 0.028
FIR	1 - 8 Hz	All speaking: (S) + (S|B) + Silence	0.040 ± 0.009

Supplementary Table 2: Correlations values for the different combinations of filters and frequency bands. The FIR filter corresponds to the causal finite-impulse response filter implemented in our work, and described in the manuscript. The Butterworth filter is a non-causal 3rd order Butterworth filter.

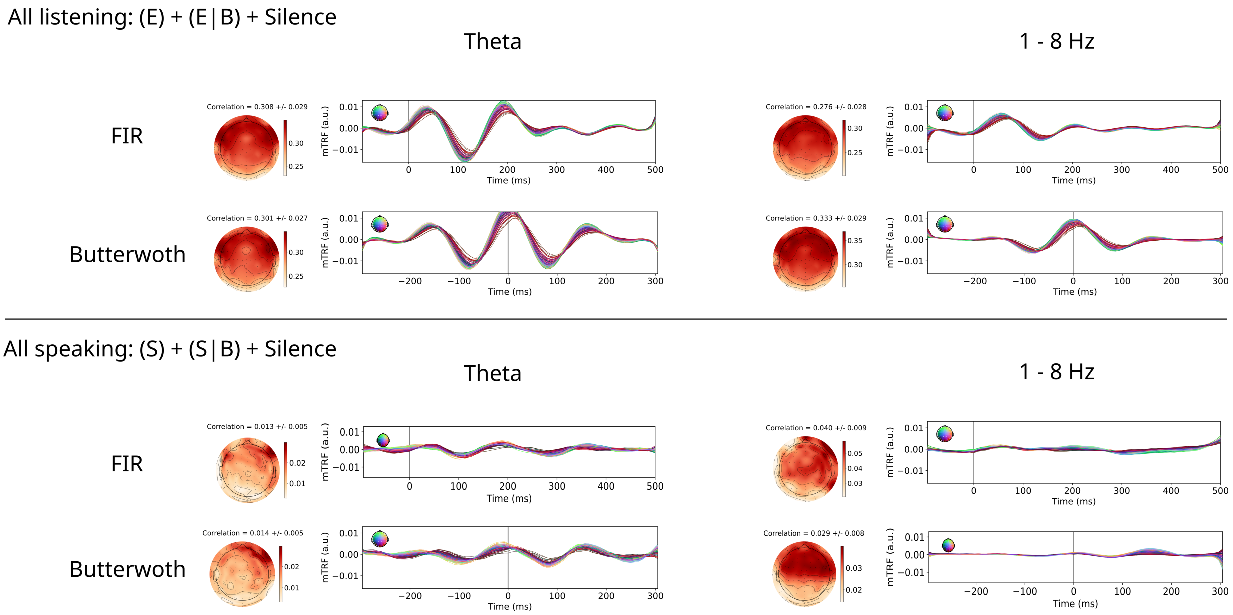

Supplementary Figure 8: Correlation values and TRFs obtained from different filtering methods, over All Listening ((E) + (E|B) + Silence) and All Speaking ((S) + (S|B) + Silence) conditions. FIR corresponds to the causal finite-impulse response filter implemented in our work, and described in the Methods section. Butterworth corresponds to a non-causal 3rd order Butterworth filter. The difference between causal and non-causal filters affects the timing on which the signal will present the most prominent activity, so the time windows in each case were determined to capture the effect on the TRFs. The time windows of TRFs corresponding to the Butterworth filters (non-causal) are from -0.3 to 0.3 s. The time windows corresponding to the causal filters are from -0.1 to 0.5 s.

From Table 2 and Figure 8 we can see that the correlation values and TRF amplitude are robust to the filters used as in all scenarios, the SIS effect seems to be present.

Supplementary Note 6: Re-referencing

In this work, the data was referenced to linked-mastoids, and the resulting mTRF are similar to those presented in,[8] which also used mastoids as reference. We repeated the analysis for the spectrogram model in the Theta band for the listening (E) condition, re-referencing the EEG signal to the EEG average. The resulting mTRF looks like those presented in,[5] who used average-reference, showing a high polarization in the peaks (Fig. 9 A). However, the predictive power in central channels drops significantly (Fig. 9 B), as they are predominant in the re-referencing to the average of all channels.

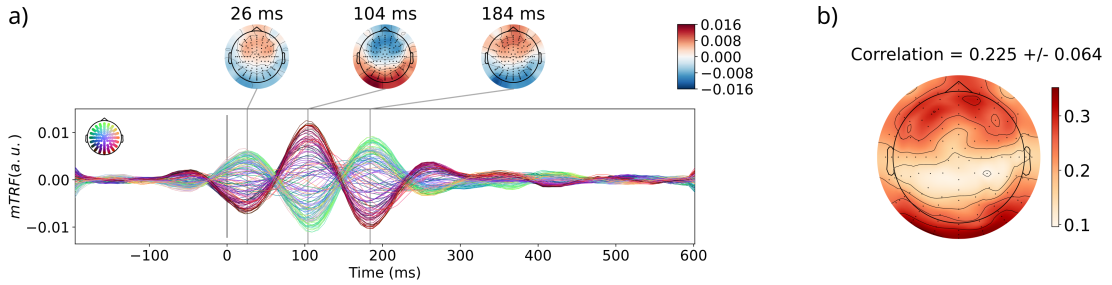

Supplementary Figure 9: Re-referencing responses to the average. Panel **A** shows the mTRF for each electrode re-referenced to the average of electrodes, and averaged across mel-bands. The position of each electrode is indicated by the scalp plot in the top-left corner. Scalp distributions at the time of the peak are presented on the top with the corresponding times. Panel **B** shows the scalp distribution of the correlation values.

Supplementary Note 7: mTRF amplitude

Repeating the encoding model analysis with the spectrogram in the Listening situation, using the whole data but partitioned in subsets of 2000 samples for the training set, the resulting mTRFs present a decreased amplitude. This could be due to the fact that now, each fold contains fewer and different samples, making it more sensitive to “bad” subsets, in which the linear relationship between the envelope and the EEG signals was null or with different patterns. Larger partitions would be more robust in this sense, making it more probable for the dynamics of the mTRFs to emerge with more samples. Averaging the model weights over these subsets, which are all averaged equally could significantly affect the resulting mTRFs

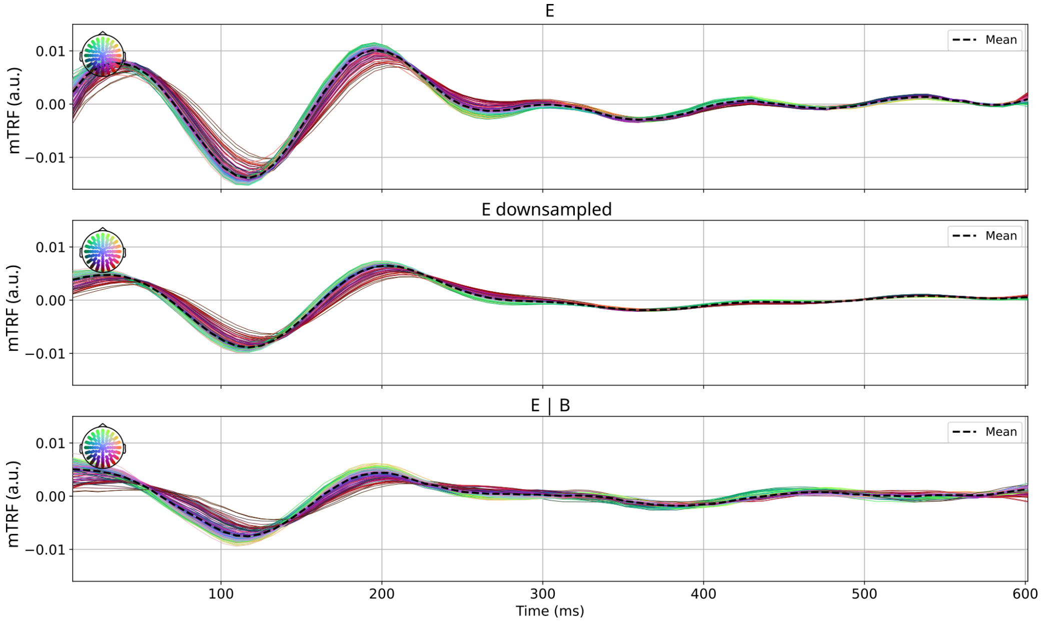

Supplementary Figure 10: TRFs obtained from the Spectrogram feature to the Theta band for the Listening condition using subsets of approximately 2000 samples for the training set.

Supplementary Note 8: False-Discovery Rate (FDR) correction

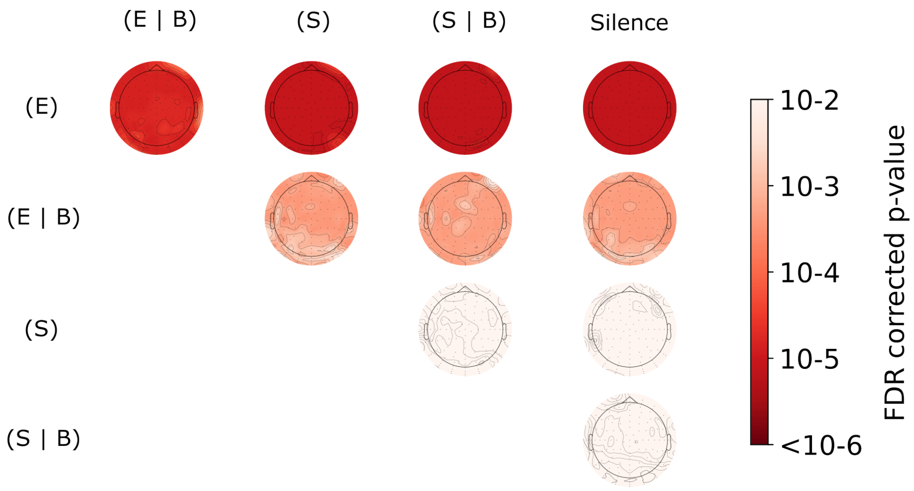

Supplementary Figure 11: Comparison between listening conditions. False-Discovery Rate (FDR) corrected p-values from a Wilcoxon signed-rank test, between the average correlation values of each electrode from different conditions, in the Theta band (N=18)

Supplementary Note 9: Alpha selection

Ridge regression has a single hyperparameter, alpha, which was tuned for each subject and feature separately. The values that this parameter takes have direct implications on the adjustment of the weights of the model, and therefore, on the predictions made by it. Very small alpha values would lead to overfitting the model to the training data.[9,10] Also, as the response from the EEG signal to the continuous stimuli is analyzed through the mTRFs, minimizing the effects of individual participants and spurious noise by smoothing the weights is very important, not only to have more interpretable and comparable mTRFs, but also, to produce a model that generalizes better.[9,10] On the other hand, with very large values, the minimization of the Equation 1 would be dominated by the penalty to the weights, instead of the fit to the data, and the predictive capacity of the model would irremediably worsen. For this reason, it is necessary to find the value of alpha that has a balance between the maximization of the correlation of the prediction, and the regularization of the adjusted weights.

$$|Y - X.w|^2 + \alpha|w|^2 \quad (1)$$

A search was made for parameters that maximize the performance of the model, that is, its predictive capacity on new data sets. This would correspond to taking a value of the regularization parameter that maximizes the correlation of the prediction on the validation set.[11,12] In order to minimize the overfitting effect, the value of the regularization parameter is slightly increased within a certain range, penalizing the values of the weights, and forcing the model to give importance to the time instants with the greatest impact on the EEG signal.

With these two criteria in mind, we automatically determine the alpha parameter in each case. From the training set, 20% was used as a validation set, and a grid search was performed between 10^-1 and 10^6 in 32 equally spaced steps on a logarithmic scale. The model was fitted and a prediction was made over the validation set for each alpha value, thus obtaining a correlation value for each prediction.

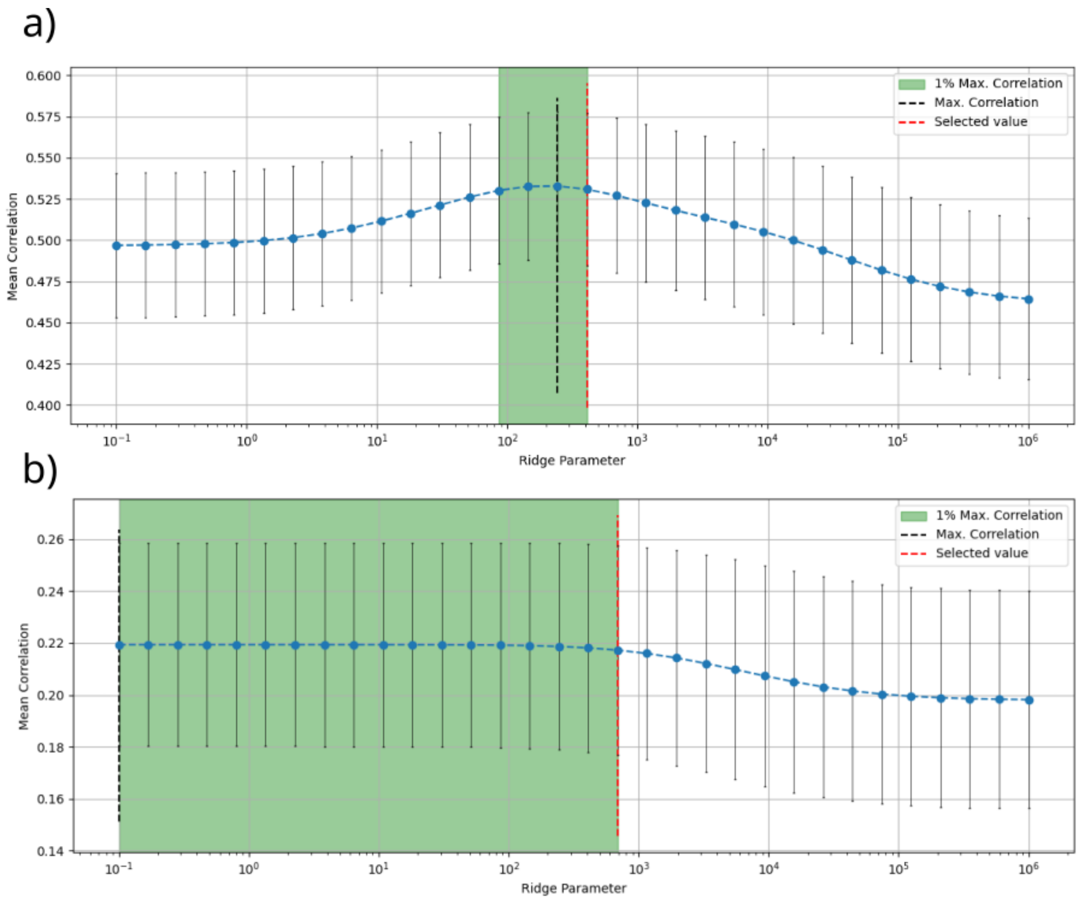

Supplementary Figure 12: A: Example of correlation values for each alpha value for one subject. The feature used as input was the spectrogram, adjusted to the Theta band of the EEG. The alpha value corresponding to the maximum correlation is shown in black dotted lines, and the interval corresponding to 1% of said correlation value is shown in green. The chosen alpha value is marked with the red dotted line. B: Example of correlation values for each alpha value for one subject. The feature used as input was the envelope, adjusted to the Theta band of the EEG. The alpha value corresponding to the maximum correlation is shown in black dotted lines, and the interval corresponding to 1% of said correlation value is shown in green. The chosen alpha value is marked with the red dotted line. The blue dots represent the mean value for all the cross-validations. The black lines in each point represent +- one standard deviation

In the first place, the average correlation of all the *folds* and channels of each subject was saved, obtained with each alpha parameter of the search, in order to choose the alpha that maximizes this value (Figure 12 A). In each case, the alpha interval was determined where the correlation value was within 99% of the maximum, in order to give a degree of freedom to take a higher alpha. The selected value is the largest alpha within the interval of maximum correlation, minimally sacrificing model prediction correlation in order to minimize collinearity effects. Furthermore, the proposed method serves to avoid cases like the one observed in Figure 12 B, where the maximum correlation is found with the smallest value of alpha. In that case, the difference in correlation with the following values is minimal, so a significantly higher alpha value can be taken, preserving the performance of the model in terms of prediction capacity, and penalizing the weights.

Supplementary Note 10: Permutations test

The input matrix for the model consisted of ($N_{samples}$) rows and ($N_{times} \times N_{features}$) columns. Each sample corresponded to an interval where the participant had been uninterruptedly listening to their partner speak for at least 0.6 seconds (condition E in our manuscript). Using overlapped sliding windows with 1 time-point step, all the valid intervals within each session were extracted (for each condition separately). Around 50,000 samples per participant for the E condition were obtained (Fig. 13 A, B). As the EEG and audio sampling rates were both 128Hz, each interval (or sample) of 0.6 seconds contained 77 time-points ($N_{times} = 77$). In the case of the spectrogram, as there are 16 frequency bands, the $N_{times} \times N_{features}$ correspond to a 77 x 16 = 1232 vector.

The permutations test was implemented by making 3000 random permutations of the input matrix. This analysis was performed for each participant, electrode and fold. The permutations consisted only in rearranging the samples, i.e. assigning the EEG interval to a different audio interval. Thus, these random permutations conserved the correlation structure between subsequent time-points (Fig. 13 C.). The evaluation set kept its samples and time ordering, as in the original data (Fig. 13 B.).

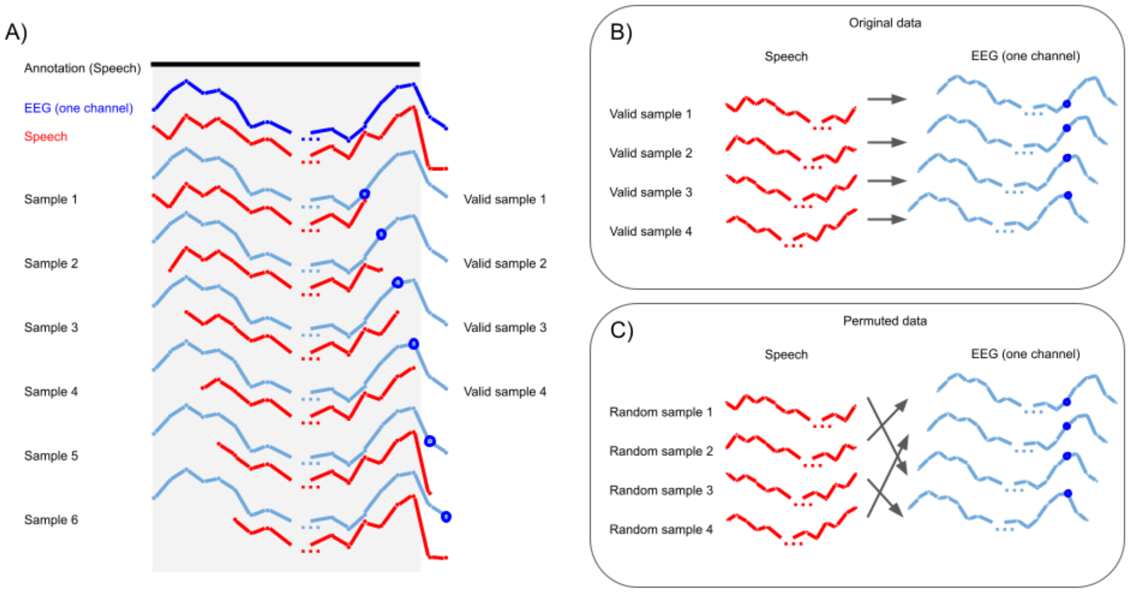

Supplementary Figure 13: Schema of the definition of samples, valid samples, and permuted samples.

Supplementary References

[1] Boersma, P. & Van Heuven, V. Speak and unspeak with praat. *Glot International* **5**, 341–347 (2001).

[2] Cole, J. *et al.* Sound, structure and meaning: The bases of prominence ratings in english, french and spanish. *Journal of Phonetics* **75**, 113–147 (2019).

[3] Jadoul, Y., Thompson, B. & De Boer, B. Introducing parselmouth: A python interface to praat. *Journal of Phonetics* **71**, 1–15 (2018).

[4] Virtanen, P. *et al.* SciPy 1.0: Fundamental Algorithms for Scientific Computing in Python. *Nature Methods* **17**, 261–272 (2020).

[5] Etard, O. & Reichenbach, T. Neural speech tracking in the theta and in the delta frequency band differentially encode clarity and comprehension of speech in noise. *Journal of Neuroscience* **39**, 5750–5759 (2019).

[6] Lalor, E. C., Power, A. J., Reilly, R. B. & Foxe, J. J. Resolving precise temporal processing properties of the auditory system using continuous stimuli. *Journal of neurophysiology* **102**, 349–359 (2009).

[7] Lalor, E. C. & Foxe, J. J. Neural responses to uninterrupted natural speech can be extracted with precise temporal resolution. *European journal of neuroscience* **31**, 189–193 (2010).

[8] Di Liberto, G. M., O'Sullivan, J. A. & Lalor, E. C. Low-frequency cortical entrainment to speech reflects phoneme-level processing. *Current Biology* **25**, 2457–2465 (2015).

[9] Crosse, M. J., Di Liberto, G. M., Bednar, A. & Lalor, E. C. The multivariate temporal response function (mtrf) toolbox: a matlab toolbox for relating neural signals to continuous stimuli. *Frontiers in human neuroscience* **10**, 604 (2016).

[10] Crosse, M. J. *et al.* Linear modeling of neurophysiological responses to speech and other continuous stimuli: methodological considerations for applied research. *Frontiers in neuroscience* **15**, 705621 (2021).

[11] Hamilton, L. S., Edwards, E. & Chang, E. F. A spatial map of onset and sustained responses to speech in the human superior temporal gyrus. *Current Biology* **28**, 1860–1871 (2018).

[12] Desai, M. *et al.* Generalizable eeg encoding models with naturalistic audiovisual stimuli. *Journal of Neuroscience* **41**, 8946–8962 (2021).
