# Supplementary material for: Speech-induced suppression during natural dialogues
Source: Commun Biol. 2024 Mar 8;7:291. doi: 10.1038/s42003-024-05945-9 (PMC10923813; doi:10.1038/s42003-024-05945-9)
Supplement: Supplementary file 3 — Reporting Summary [file 42003_2024_5945_MOESM3_ESM.pdf]

nature portfolio

Corresponding author(s): Joaquin Gonzalez

Last updated by author(s): Jan 29, 2024

Reporting Summary

Nature Portfolio wishes to improve the reproducibility of the work that we publish. This form provides structure for consistency and transparency in reporting. For further information on Nature Portfolio policies, see our Editorial Policies and the Editorial Policy Checklist.

Statistics

For all statistical analyses, confirm that the following items are present in the figure legend, table legend, main text, or Methods section.

n/a	Confirmed	
☐	☒	The exact sample size (n) for each experimental group/condition, given as a discrete number and unit of measurement
☐	☒	A statement on whether measurements were taken from distinct samples or whether the same sample was measured repeatedly
☐	☒	The statistical test(s) used AND whether they are one- or two-sided<br>*Only common tests should be described solely by name; describe more complex techniques in the Methods section.*
☐	☒	A description of all covariates tested
☐	☒	A description of any assumptions or corrections, such as tests of normality and adjustment for multiple comparisons
☐	☒	A full description of the statistical parameters including central tendency (e.g. means) or other basic estimates (e.g. regression coefficient) AND variation (e.g. standard deviation) or associated estimates of uncertainty (e.g. confidence intervals)
☐	☒	For null hypothesis testing, the test statistic (e.g. F, t, r) with confidence intervals, effect sizes, degrees of freedom and P value noted<br>*Give P values as exact values whenever suitable.*
☒	☐	For Bayesian analysis, information on the choice of priors and Markov chain Monte Carlo settings
☒	☐	For hierarchical and complex designs, identification of the appropriate level for tests and full reporting of outcomes
☒	☐	Estimates of effect sizes (e.g. Cohen's d, Pearson's r), indicating how they were calculated

Our web collection on statistics for biologists contains articles on many of the points above.

Software and code

Policy information about availability of computer code

Data collection	Data collection code was written in java, and is available with the rest of preprocessing and analysis codes in https://github.com/jegonza66/SIS-during-natural-dialogue.
Data analysis	Data preprocessing was performed using EEGLAB toolbox (Version 13.4.3b) in Matlab 2015b. The analyses presented in the manuscript were performed on python using MNE 0.24.0 and SciPy 1.7.1 libraries. The codes are available at https://github.com/jegonza66/SIS-during-natural-dialogue.

For manuscripts utilizing custom algorithms or software that are central to the research but not yet described in published literature, software must be made available to editors and reviewers. We strongly encourage code deposition in a community repository (e.g. GitHub). See the Nature Portfolio guidelines for submitting code & software for further information.

Data

Policy information about availability of data

All manuscripts must include a data availability statement. This statement should provide the following information, where applicable:

- Accession codes, unique identifiers, or web links for publicly available datasets
- A description of any restrictions on data availability
- For clinical datasets or third party data, please ensure that the statement adheres to our policy

Speech data was part of the UBA Games Corpus, which was already released in an institutional public repository (https://ri.conicet.gov.ar/handle/11336/191235) [Gravano2023]. EEG data can be found in https://doi.org/10.6084/m9.figshare.22647313.

Research involving human participants, their data, or biological material

Policy information about studies with human participants or human data. See also policy information about sex, gender (identity/presentation), and sexual orientation and race, ethnicity and racism.

Reporting on sex and gender	Twenty participants (10 men and 10 women) took part in the experiment. No sex or gender-based analysis was performed on this work because it was out of the scope of the study.
Reporting on race, ethnicity, or other socially relevant groupings	All the participants were reached within the university. They were undergraduate and graduate students. No race or ethnicity-based analysis was performed on this work because it was out of the scope of the study.
Population characteristics	The only population characteristics we collected was sex and age only for reporting purposes. All participants were native Spanish speakers.
Recruitment	Participants were contacted by email. They were asked for neurological / neuropsychological diagnostics and use psychoactive drugs, their age and if they were native Spanish speakers. These were the only inclusion criteria we applied.
Ethics oversight	All the experiments described in this paper were reviewed and approved by the ethics committee: Comité de Ética del Centro de Educación Médica e Investigaciones Clínicas "Norberto Quirno" (CEMIC) and qualified by the Department of Health and Human Services (HHS, USA): IRb00001745 - IORG 0001315 (Protocol 435). All participants provided written informed consent in agreement with the Helsinki declaration.

Note that full information on the approval of the study protocol must also be provided in the manuscript.

Field-specific reporting

Please select the one below that is the best fit for your research. If you are not sure, read the appropriate sections before making your selection.

☒ Life sciences ☐ Behavioural & social sciences ☐ Ecological, evolutionary & environmental sciences

For a reference copy of the document with all sections, see nature.com/documents/nr-reporting-summary-flat.pdf

Life sciences study design

All studies must disclose on these points even when the disclosure is negative.

Sample size

Twenty participants took part in the study (10 female, 10 male), ages ranging between 19 and 43 years (M = 26.4, SD = 6.3), and 18 were right-handed. All subjects were native speakers of Argentine Spanish, and lived in the Buenos Aires area at the time of the study. They were undergraduate and graduate students, and they did not know their partner in the dialogue session before the study.
The data was originally recorded to study neural correlates of turn-taking cues during natural dialogues. The sample size was decided based on previous studies on speech turn-taking cues on a similar corpus in English [Gravano & Hirschberg, 2009; 2011], that are now replicated in Spanish [Brusco, et al., 2020; Brusco & Gravano, 2023]. The data was sufficient for the current study based on other similar studies, moreover, many of the analyses were performed on a single-subject basis and then summarized.

References:
Brusco, P., Vidal, J., Beňuš, Š., & Gravano, A. (2020). A cross-linguistic analysis of the temporal dynamics of turn-taking cues using machine learning as a descriptive tool. Speech Communication, 125, 24-40.
Brusco, P., & Gravano, A. (2023). Automatic offline annotation of turn-taking transitions in task-oriented dialogue. Computer Speech & Language, 78, 101462.
Gravano, A., & Hirschberg, J. (2009, September). Turn-yielding cues in task-oriented dialogue. In Proceedings of the SIGDIAL 2009 Conference (pp. 253-261).
Gravano, A., & Hirschberg, J. (2011). Turn-taking cues in task-oriented dialogue. Computer Speech & Language, 25(3), 601-634.

Data exclusions

One session (involving two participants) was excluded due to extremely bad EEG data quality. This was assessed by eye on the day of data collection and the EEG data were not processed at all. Nevertheless, the audio files were available in Gravano, et al. (2023).

References:

Gravano, Agustin; Kamienkowski, Juan Esteban; Brusco, Pablo; (2023): UBA Games Corpus. Consejo Nacional de Investigaciones Científicas y Técnicas. (dataset). http://hdl.handle.net/11336/191235

Replication	The temporal response function (TRF) to auditory features were assessed on a single-subject basis. The average correlation of the TRFs between participants was between 0.65 and 0.70 for different features. The performance of the models was significant for several channels in every participant, also assessed on a single-subject basis. Thus, the results could be replicated in every participant.
Randomization	The participants were not allocated in groups. The couples that share a recording session (dialogues) were assigned based on their schedules and on a first-come first-serve basis. We only verified that none of the participants knew each other before the experiment.
Blinding	All the analyses were performed within participants, and no grouping was made. At the moment of data collection, participants were blind to the purposes of the experiment. The pre-analysis was performed considering all the data of each participant together, without separation of conditions. Moreover, the conditions that were compared in the manuscript are intervals of speech and silence of one or both participants, that were naturally intermixed in the dialogues. The analysis for the final comparison was exactly the same for any condition and did not involve a decision on the experimenters side.

Reporting for specific materials, systems and methods

We require information from authors about some types of materials, experimental systems and methods used in many studies. Here, indicate whether each material, system or method listed is relevant to your study. If you are not sure if a list item applies to your research, read the appropriate section before selecting a response.

Materials & experimental systems

n/a	Involved in the study	
☒	☐	Antibodies
☒	☐	Eukaryotic cell lines
☒	☐	Palaeontology and archaeology
☒	☐	Animals and other organisms
☒	☐	Clinical data
☒	☐	Dual use research of concern
☒	☐	Plants

Methods

n/a	Involved in the study	
☒	☐	ChIP-seq
☒	☐	Flow cytometry
☒	☐	MRI-based neuroimaging

Plants

Seed stocks	N/A
Novel plant genotypes	N/A
Authentication	N/A
